# CD40 forward signalling is a physiological regulator of early sensory axon growth

**DOI:** 10.1242/dev.176495

**Published:** 2019-09-15

**Authors:** Laura Howard, Thomas G. McWilliams, Sean Wyatt, Alun M. Davies

**Affiliations:** School of Biosciences, Cardiff University, Museum Avenue, Cardiff CF10 3AT, UK

**Keywords:** TNF superfamily, CD40, CD40L, Sensory axons, Tissue innervation, Mouse

## Abstract

Multiple members of the tumour necrosis factor superfamily (TNFSF) regulate the growth and branching of neural processes late in development, when neurons are establishing and refining connections. Here, we present the first evidence that a TNFSF member acts much earlier in development, when axons are growing to their targets. CD40L transiently enhanced axon growth from embryonic mouse DRG neurons cultured at this early stage. Early spinal nerves of embryos lacking the CD40L receptor (*Cd40*^−/−^ mice) were significantly shorter *in vivo* than those of *Cd40*^+/+^ littermates. CD40L was synthesized in early DRG targets and was co-expressed with CD40 in early DRG neurons. Whereas CD40L enhanced early axon growth independently of neurotrophins, disruption of a CD40L/CD40 autocrine loop impaired early neurotrophin-promoted axon growth. In marked contrast to the widespread regulation of axon and dendrite growth by CD40L reverse signalling later in development, CD40-Fc, which activates reverse signalling, had no effect on early sensory axon growth. These results suggest that CD40 forward signalling is a novel physiological regulator of early axon growth that acts by target-derived and autocrine mechanisms.

## Abstract (precis)

**Summary:** CD40L, a novel physiological regulator of early sensory axon growth at the stage when sensory axons are growing to their targets, activates CD40 forward signalling by target-derived and autocrine mechanisms.

## INTRODUCTION

The growth and elaboration of neural processes in the developing nervous system is regulated by a variety of extracellular signalling proteins that act on developing neurons. Much of our understanding of the principles by which such signals operate has been gained by studying neurons of the peripheral nervous system (PNS). The most extensively characterized signals are those provided by members of the nerve growth factor (NGF) family of neurotrophins, which include NGF, brain derived neurotrophic factor (BDNF), neurotrophin 3 (NTF3) and neurotrophin 4 (NTF4). These secreted proteins are expressed in a variety of tissues and not only sustain the survival of different kinds of neurons but also promote the growth and ramification of axons in target tissues (Davies, 2003; Dekkers and Barde, 2013; Glebova and Ginty, 2005; Huang and Reichardt, 2001). Because of this, neurotrophins play key roles in regulating the number of neurons that survive during development and in controlling tissue innervation density.

Work over the past decade has revealed that multiple members of the tumour necrosis factor superfamily (TNFSF) are physiological regulators of axon growth without affecting neuronal survival. The 19 members of this superfamily have been most extensively characterized in the immune system (Hehlgans and Pfeffer, 2005; Šedý et al., 2014). They are synthesized as type 2 membrane glycoproteins that bind to one or more members of the TNF receptor superfamily (TNFRSF). They exert their actions as membrane-bound ligands and as soluble ligands following proteolytic cleavage and release of the extracellular receptor-binding domain from the cell membrane (Grivennikov et al., 2006; Hehlgans and Pfeffer, 2005). In the developing PNS, it has been reported that members of the TNFSF act on neurons well after they become dependent on neurotrophins for survival, during the stage when their axons are ramifying and refining terminations in target tissues. Whereas some TNFSF members enhance axon growth (McWilliams et al., 2017; O'Keeffe et al., 2008; Osório et al., 2014), others reduce the axon growth-promoting actions of neurotrophins (Gavalda et al., 2009; Gutierrez et al., 2013). They act on particular kinds of neurons either in a target-derived manner or via autocrine signalling loops.

In addition to functioning as ligands, several membrane-integrated TNFSF members can act as reverse signalling receptors for the TNFRSF members to which they bind. TNFSF reverse signalling has been widely documented in the immune system (Sun and Fink, 2007) and has recently been shown to regulate the growth of neural processes in the developing nervous system. TNFR1 (TNFRSF1A)-activated TNF reverse signalling enhances the growth of sympathetic and sensory axons and promotes tissue innervation (Kisiswa et al., 2013, 2017; Wheeler et al., 2014). BCMA (TNFRSF17)-activated TWE-PRIL (TNFSF12-TNFSF13) reverse signalling suppresses sympathetic axon growth and tissue innervation (Howard et al., 2018). CD40-activated CD40L (CD40LG) reverse signalling is extensively involved in regulating both axon and dendrite growth in the developing PNS and CNS. It enhances axon growth from a subset of sympathetic neurons and hippocampal pyramidal neurons and selectively regulates the sympathetic innervation of particular tissues *in vivo* (Carriba and Davies, 2017; McWilliams et al., 2015). CD40-activated CD40L reverse signalling is a major *in vivo* regulator of dendrite growth and arborization in the CNS, promoting the growth and elaboration of excitatory neuron dendrites and suppressing the growth and elaboration of inhibitory neuron dendrites (Carriba and Davies, 2017).

Here, we have focused on the sensory neurons of developing mouse dorsal root ganglia (DRG). These neurons are derived from the neural crest and extend axons to central targets in the spinal cord and peripheral targets in developing skin and a great variety of other tissues (Lallemend and Ernfors, 2012). Newly differentiated sensory neurons survive and extend axons independently of neurotrophins when their axons are growing to their targets and become dependent on neurotrophins for survival as their axons reach their targets (Davies et al., 1987; Vogel and Davies, 1991). Survival dependence on particular neurotrophins becomes restricted to functionally distinct subsets of DRG neurons as development progresses (Ernsberger, 2009). Although subsets of neurotrophin-dependent DRG neurons subsequently acquire survival responses to members of glial cell-derived neurotrophic factor (GDNF) in late fetal development *in vitro* (Baudet et al., 2000), these factors play no role in sustaining the survival of all but a very small functional subset of DRG neurons *in vivo* (Fleming et al., 2015). There is evidence, however, that the GDNF family play a role in specifying functional subsets of DRG neurons (Ernsberger, 2008, 2009; Lallemend and Ernfors, 2012).

In this publication, we report that CD40L-activated CD40 forward signalling is a physiological regulator of axon growth, but not neuron survival, for developing DRG neurons. However, in contrast to all other TNFSF members, which regulate axon growth during a late developmental window well after axons have reached their target tissues and are ramifying in and refining terminations in these tissues, CD40 forward signalling acts very early and transiently in development when DRG axons are growing to their targets.

## RESULTS

### CD40L enhances early *in vitro* DRG axon growth independently of neurotrophins

**Fig. 1.**
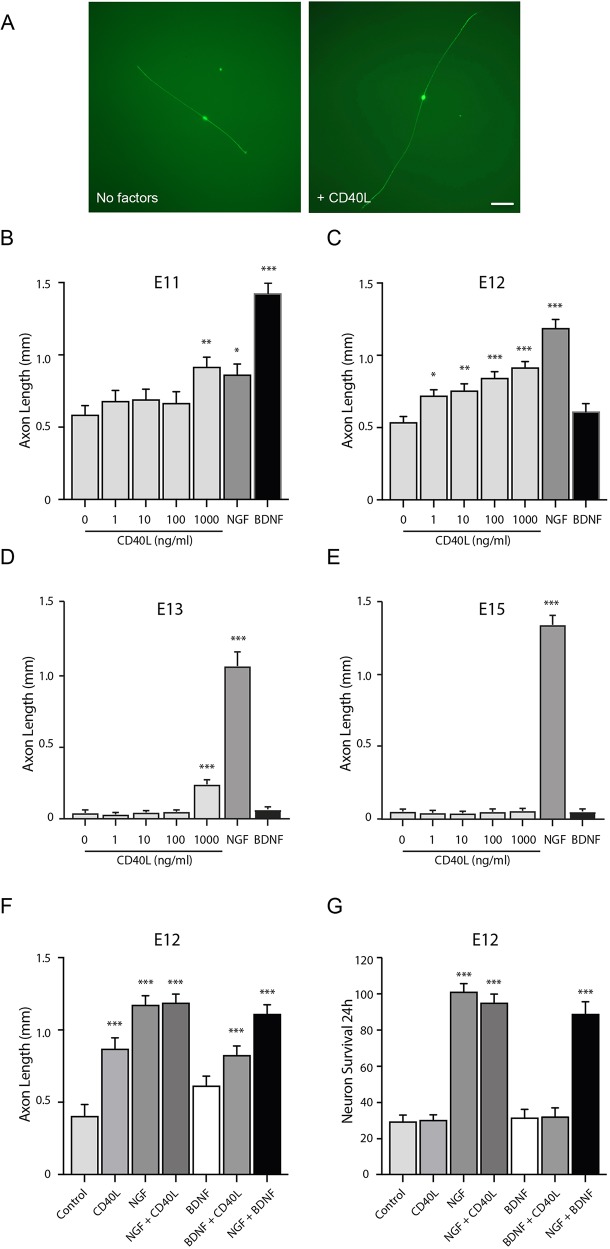
**CD40L enhances axon growth from early DRG neurons.** (A) Representative images of E12 DRG neurons cultured for 24 h with and without 100 ng/ml CD40L. Scale bar: 100 µm. (B-E) Length of axons growing from E11 (B), E12 (C), E13 (D) and E15 (E) DRG neurons cultured for 24 h with either different concentrations of CD40L, 10 ng/ml NGF, 10 ng/ml BDNF or no factors. (F) Length of axons growing from E12 DRG neurons cultured for 24 h with either 100 ng/ml CD40L, 10 ng/ml NGF, 10 ng/ml BDNF, NGF plus BDNF, CD40L plus NGF, CD40L plus BDNF or no added factors. All cultures (A-F) received 25 µM Boc-D-FMK. Data are mean±s.e.m. of >50 neurons per condition obtained from three separate experiments. (G) Percent survival of E12 DRG neurons after 24 h in cultures without Boc-D-FMK, normalized to 100% survival for NGF-supplemented cultures. Data are mean±s.e.m. from three separate experiments. *P<0.05, **P<0.01, ***P<0.001; one-way ANOVA with Bonferroni correction, comparison with control.

To study the potential effect of CD40L on axon growth from developing sensory neurons, we established low-density dissociated cultures of embryonic mouse DRG neurons over a range of ages beginning at embryonic day (E)11, the earliest age at which DRG can be reliably dissected from embryos. To prevent neuronal apoptosis, we included the pan-caspase inhibitor Boc-D-FMK in the culture medium. Neurons in these cultures exhibited the characteristic bipolar morphology of early sensory neurons (Fig. 1A). In E11 cultures, when the earliest DRG axons are growing to their targets *in vivo*, CD40L significantly enhanced axon growth at only the highest concentration used (1 µg/ml) (Fig. 1B). In E12 cultures, the neurons exhibited enhanced sensitivity to CD40L, with a significant increase of axon length with levels of CD40L as low as 1 ng/ml (Fig. 1C). This response to CD40L was, however, very transient. By E13, the neurons had lost responsiveness to CD40L at all but the highest concentration used (Fig. 1D), and at later ages no response to CD40L was observed (Fig. 1E). Although CD40L is the main ligand for CD40, Hsp70 (Hspa1b) can also function as a CD40 ligand that modulates CD40 activity (Wang et al., 2001; Becker et al., 2002). However, Hsp70 at concentrations ranging from 1 ng/ml to 1 µg/ml had no effect on early sensory axon growth (not shown), suggesting that Hsp70 does not modulate early sensory axon growth.

Neural crest-derived sensory neurons, such as DRG and trigeminal ganglion neurons, respond to neurotrophins in development. In cultures set up at sequential stages of development, neurons initially survive and extend axons independently of neurotrophins before responding to BDNF. Shortly after, they begin responding to NGF with the arrival of the earliest peripheral axons at their cellular targets (Buchman and Davies, 1993; Davies et al., 1987). This temporal sequence of neurotrophin responsiveness is due in part to the earlier birth of BDNF-responsive neurons and partly because some neurons switch responsiveness from BDNF to NGF early in development (Enokido et al., 1999).

To compare the timing of CD40L responsiveness with neurotrophin responsiveness, we studied the effect of BDNF, NGF and CD40L in cultures set up at different ages. In E11 cultures, BDNF promoted a very marked axon growth response (Fig. 1B). By E12 the response to BDNF had greatly decreased and a marked response to NGF had become apparent (Fig. 1C). Although the magnitude of the axon growth response to CD40L had also increased by E12, this response was less than that elicited by NGF, even at the highest concentration of CD40L used (Fig. 1C). In E12 cultures, there was no additive effect of saturating concentrations of CD40L with either BDNF or NGF (Fig. 1F), suggesting that the neuronal populations that respond to neurotrophins and CD40L were overlapping.

### CD40L does not affect DRG neuron survival

In the absence of Boc-D-FMK, the majority of E12 neurons underwent apoptosis in control cultures (no added factors) by 24 h incubation (Fig. 1G). In contrast to the pronounced survival response of the majority of neurons to NGF, there was no significant difference in survival between control cultures and CD40L-supplemented cultures (Fig. 1G). There was no additional survival in cultures treated with CD40L plus NGF, compared with NGF-treated cultures, and there was no significant difference in survival between cultures treated with CD40L plus BDNF and control cultures (Fig. 1G). Taken together, these findings suggest that CD40L enhances the growth of axons from early DRG neurons independently of neurotrophins at the stage when axons are growing to their targets *in vivo*, without affecting neuron survival.

### CD40L enhances early axon growth by CD40-mediated forward signalling *in vitro*

**Fig. 2.**
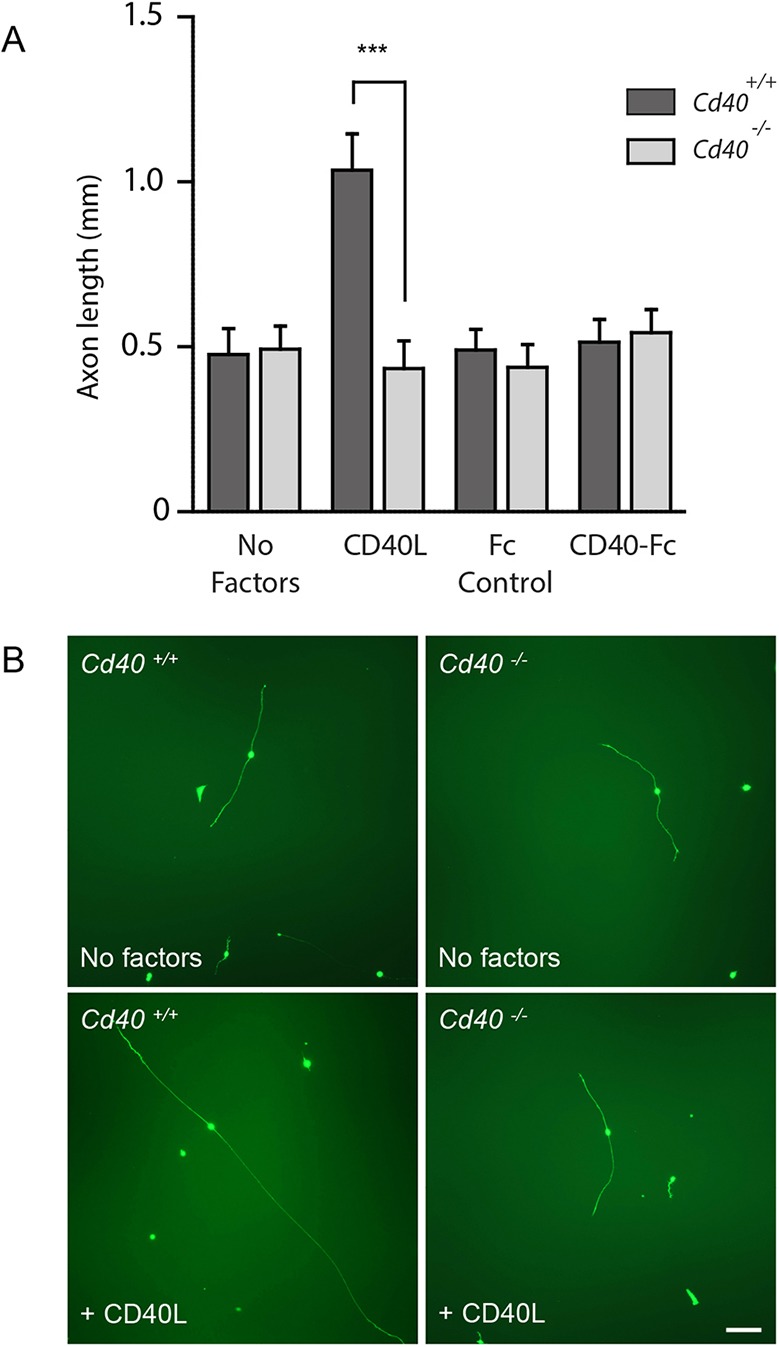
**Deletion of the *Cd40* gene eliminates the CD40L axon growth response.** (A) Axon length of E12 DRG neurons of *Cd40*^−/−^ and *Cd40*^+/+^ littermates cultured for 24 h with 25 µM Boc-D-FMK. In addition to controls (which received no additional reagents), the cultures received either 100 ng/ml CD40L, 1 µg/ml CD40-Fc or 1 µg/ml control Fc protein. Data are mean±s.e.m. of >50 neurons per condition obtained from three separate experiments. ***P<0.001, *t*-test. (B) Representative images of E12 DRG neurons of *Cd40*^−/−^ and *Cd40*^+/+^ littermates after 24 h in cultures supplemented with 25 µM Boc-D-FMK and either with no added reagents (controls) or 100 ng/ml CD40L. Scale bar: 100 µm.

To clarify the manner by which CD40L stimulates axon growth from early DRG neurons, we studied axon growth from E12 DRG neurons cultured from *Cd40*^−/−^ embryos and *Cd40*^+/+^ littermates in the absence of NGF but with Boc-D-FMK to sustain survival. The marked axon growth response of neurons cultured from *Cd40*^+/+^ mice to soluble CD40L was completely eliminated in neurons cultured from *Cd40*^−/−^ mice (Fig. 2). There was no significant difference in axon length between neurons cultured from *Cd40*^−/−^ mice grown with and without CD40L (Fig. 2A). A soluble CD40-Fc chimera (in which the extracellular domain of CD40 is linked to the Fc part of human IgG1) that activates CD40L-mediated reverse signalling (Carriba and Davies, 2017; McWilliams et al., 2015) did not significantly affect axon growth. There was no significant difference in axon length between neurons that were treated with a concentration of CD40-Fc (1 µg/ml) that has previously been shown to activate reverse signalling in neurons and neurons treated with the same concentration of Fc control protein (Fig. 2A). To be sure of this point, we performed an extensive dose-response analysis using CD40-Fc at concentrations ranging from 0.1 to 10 µg/ml. CD40-Fc did not significantly affect axon growth at any concentration within this range (data not shown). The appearance of representative DRG neurons cultured from E12 *Cd40*^−/−^ and *Cd40*^+/+^ embryos grown for 24 h with and without CD40L are shown in Fig. 2B. Taken together, these results suggest that CD40L promotes early sensory axon growth by activating CD40-mediated forward signalling.

### Decreased *in vivo* length of spinal nerves of *Cd40*^−/−^ embryos

**Fig. 3.**
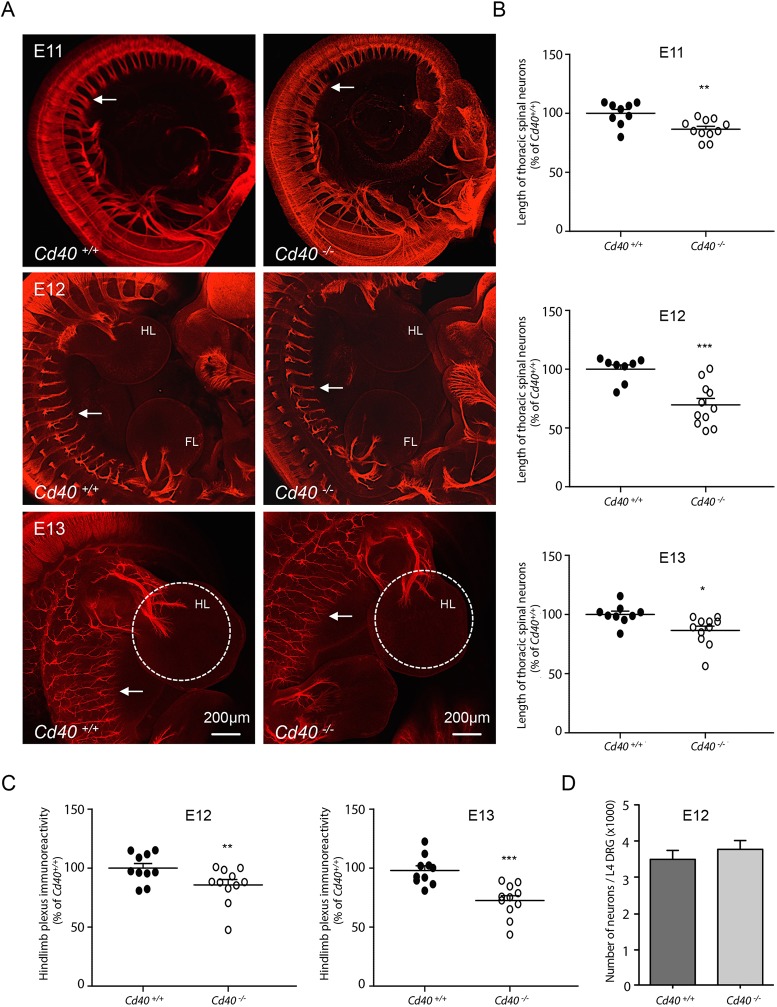
**Decreased *in vivo* length of spinal nerves of *Cd40*^−/−^ embryos.** (A) Representative iDISCO images of E11, E12 and E13 embryos of *Cd40*^−/−^ and *Cd40*^+/+^ littermates labelled with anti-βIII-tubulin. Arrows indicate the distal tips of spinal nerves. FL, fore limb; HL, hind limb. The standardized regions in which hind limb plexus βIII-tubulin immunoreactivity was assessed in E13 embryos is indicated by the dashed circles. Scale bars: 200 µm. (B) Scatter plots of the measurements between the distal tips of mid-thoracic spinal nerves and the point at which the corresponding spinal roots contact the spinal cord, normalized to 100% for the *Cd40*^+/+^ mean at each age. Each point represents the mean measurement from individual embryos at E11, E12 and E13. (C) Scatter plots of hind limb plexus βIII-tubulin immunoreactivity normalized to 100% for the *Cd40*^+/+^ mean at E12 and E13. (D) Bar chart of the numbers of neurons in the L4 DRG of E12 *Cd40*^−/−^ and *Cd40*^+/+^ littermates (*n*=4 embryos per genotype). Data are mean±s.e.m. *P<0.05, **P<0.01, ***P<0.001; *t*-test, comparison between *Cd40*^−/−^ and *Cd40*^+/+^ embryos at each age.

To ascertain the physiological significance of our *in vitro* observations, we used immunolabelling-enabled three-dimensional imaging of solvent-cleared organs (iDISCO) (Renier et al., 2014) to visualize and compare spinal nerves in *Cd40*^−/−^ embryos and *Cd40*^+/+^ littermates. Axons were labelled using an antibody against the neuron-specific marker βIII-tubulin (Tubb3). Embryos were studied at three stages of development: E11, E12 and E13. Although the gross anatomy of the spinal nerves was similar in *Cd40*^−/−^ and *Cd40*^+/+^ embryos at each age (Fig. 3A), the spinal nerves emerging from DRG were significantly shorter in *Cd40*^−/−^ embryos compared with *Cd40*^+/+^ embryos. Nerve length was most accurately quantified by measuring the length between the distal tips of segmental spinal nerves and the point at which the central rami of the DRG contact the spinal cord for the same lower thoracic DRG. Blind quantification revealed highly significant reductions in nerve length in *Cd40*^−/−^ embryos compared with *Cd40*^+/+^ embryos at each age (Fig. 3B). To provide an additional level of quantification, we compared the overall size of the hind limb plexus in both genotypes at E12 and E13 when the plexus starts forming by quantifying the intensity of βIII-tubulin immunolabelling in a standardized region centred on the plexus. This analysis revealed highly significant reductions in immunolabelling in *Cd40*^−/−^ embryos compared with *Cd40*^+/+^ embryos at both ages (Fig. 3C).

To exclude the possibility that differences in nerve length were secondary to differences in the number of neurons in the DRG of *Cd40*^−/−^ and *Cd40*^+/+^ embryos, we quantified the number of neurons in a standard DRG (L4) in E12 *Cd40*^−/−^ and *Cd40*^+/+^ littermates. This analysis revealed no significant differences in neuron number between *Cd40*^−/−^ and *Cd40*^+/+^ mice (Fig. 3D). This result is consistent with the lack of effect of CD40L on the survival of DRG neurons (Fig. 1G). Taken together, our finding that *Cd40*^−/−^ embryos exhibit a phenotype *in vivo* that matches the effect of soluble CD40L on early DRG axon growth *in vitro* demonstrates the physiological relevance of these findings.

Although *Cd40*^−/−^ and *Cd40*^+/+^ embryos were anatomically similar, to exclude the possibility that differences in size between *Cd40^+/+^ and Cd40^−/−^* embryos could account for differences in sensory nerve length, we measured the length of a defined region of the sympathetic chain in E11 *Cd40^+/+^ and Cd40^−/−^* embryos. In contrast to sensory fibre length, there was no significant difference between sympathetic chain length in *Cd40^+/+^ and Cd40^−/−^* embryos (Fig. S1).

### Expression of *Cd40l* mRNA in DRG and peripheral targets

**Fig. 4.**
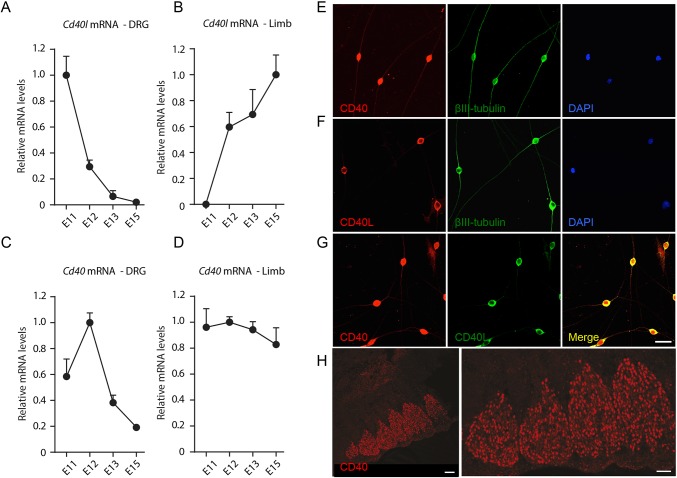
**Expression of CD40 and CD40L.** (A-D) Graphs of the levels of *Cd40l* mRNA (A,B) and *Cd40* mRNA (C,D) in the DRG (A,C) and hind limbs (B,D) of E11, E12, E13 and E15 embryos relative to the geometric mean of reference mRNAs for *Gapdh*, *Sdha* and *Hprt1*. The data are normalized to 1.0 for the highest level of expression of *Cd40l* mRNA and *Cd40* mRNA for each age series. (E-G) Representative photomicrographs of low-density E12 DRG neuron cultures double labelled with either anti-CD40 and anti-βIII-tubulin (E), anti-CD40L and anti-βIII-tubulin (F) or anti-CD40 and anti-CD40L (G). All cultures were also stained with the nuclear marker DAPI. Scale bar: 20 µm. (H) Representative low and high power immunohistochemical images of sectioned E12 DRG stained with anti-CD40 antibody. Scale bars: 100 µm (left) and 50 µm (right).

Neurons could potentially obtain CD40L *in vivo* locally and/or from their targets. To address these possibilities, we used reverse transcription-qPCR (RT-qPCR) to quantify the relative levels of *Cd40l* mRNA in DRG and DRG targets (hind limb buds) dissected from embryos at stages during the period when DRG axons are emerging from DRG and are growing toward and reaching their targets *in vivo* (E11 to E15). Because *Cd40l* mRNA is difficult to detect by conventional qPCR, we used a nested primer approach. *Cd40l* mRNA was clearly detected in DRG at E11. Relative to the geometric means of mRNAs encoding three housekeeping proteins (glyceraldehyde phosphate dehydrogenase, succinate dehydrogenase and hypoxanthine phosphoribosyltransferase 1) the level of *Cd40l* mRNA in DRG decreased markedly with age, becoming almost undetectable by E15 (Fig. 4A). In contrast, *Cd40l* mRNA was undetectable in hind limb buds at E11 (Fig. 4B). It was first detected at E12 and its relative level increased at older ages to reach a maximum at E15 (Fig. 4B). These findings suggest that CD40L is first synthesized locally in DRG when axons are starting to grow to their targets. At later ages, as DRG axons reach their targets, CD40L synthesis begins and increases in target tissues and decreases locally in DRG.

### Expression of *Cd40* mRNA in DRG and peripheral targets

We have shown that CD40L-activated CD40-mediated forward signalling enhances DRG axon growth during a brief period of early development. To investigate whether the timing of CD40L responsiveness is controlled by receptor expression, we used RT-qPCR to measure the level of *Cd40* mRNA in early DRG as an indirect measure of CD40 expression. *Cd40* mRNA was detected in early DRG at all stages studied, with a clear peak in expression at E12 followed by a marked decrease (Fig. 4C). The peak in *Cd40* mRNA expression coincides with maximum responsiveness of cultured DRG to CD40L, suggesting that the timing of CD40 expression may influence the timing of CD40L responsiveness. For completeness we also measured the relative levels of *Cd40* mRNA in limb buds. Surprisingly, *Cd40* mRNA was detectable at each age studied, with a gradual decrease in expression with age (Fig. 4D).

### Immunochemical localization of CD40 and CD40L proteins

The expression of *Cd40l* and *Cd40* mRNAs in early DRG raised the question of which cells express CD40L and CD40 and the cellular distribution of these molecules. We visualized the location of CD40 and CD40L using immunocytochemistry in dissociated cultures of E12 DRG neurons. The specificity of the anti-CD40 antibody was demonstrated by the elimination of CD40 immunofluorescence in cultures established from *Cd40^−/−^* embryos (Fig. S2). Although early DRG comprise predominantly neurons, we were particularly interested in ascertaining whether CD40 and CD40L are expressed in different subpopulations or are co-expressed. Double labelling with the neuronal marker anti-βIII-tubulin revealed that virtually all neurons expressed CD40 (94.6±2.4%, data are mean±s.e.m.) (Fig. 4E) and CD40L (92.2±3.2%) (Fig. 4F). This suggests that neurons co-express CD40 and CD40L. To formally demonstrate this, we double labelled cells with anti-CD40L and anti-CD40. These studies showed that virtually all cells were labelled by both anti-CD40L and anti-CD40 (Fig. 4G), suggesting that early DRG neurons co-express CD40 and CD40L. This in turn raised the possibility of CD40L/CD40 autocrine signalling in early DRG neurons.

The distribution of CD40 immunolabelling and CD40L immunolabelling was noticeably different on early DRG neurons. CD40 immunolabelling was clear both at the cell body of the neurons and along their axons (Fig. 4E). In contrast, CD40L immunolabelling was clearly evident at the cell body, but was either very low or negligible along the axons (Fig. 4F). This pattern is opposite to that observed on cultured sympathetic neurons using the same antibodies, where CD40L immunolabelling is clear both at the cell body and along axons and CD40 immunolabelling is clear at the cell body but very low or negligible along axons (McWilliams et al., 2015). The significance of these different distributions is unclear.

We performed immunohistochemical localization studies on sectioned embryonic DRG to compliment the above immunocytochemical studies. Although we were unable to find an anti-CD40L antibody that works reliably on sections, anti-CD40 clearly labelled neurons in sections of E12 DRG (Fig. 4H).

### CD40L/CD40 autocrine signalling contributes to neurotrophin-promoted axon growth

**Fig. 5.**
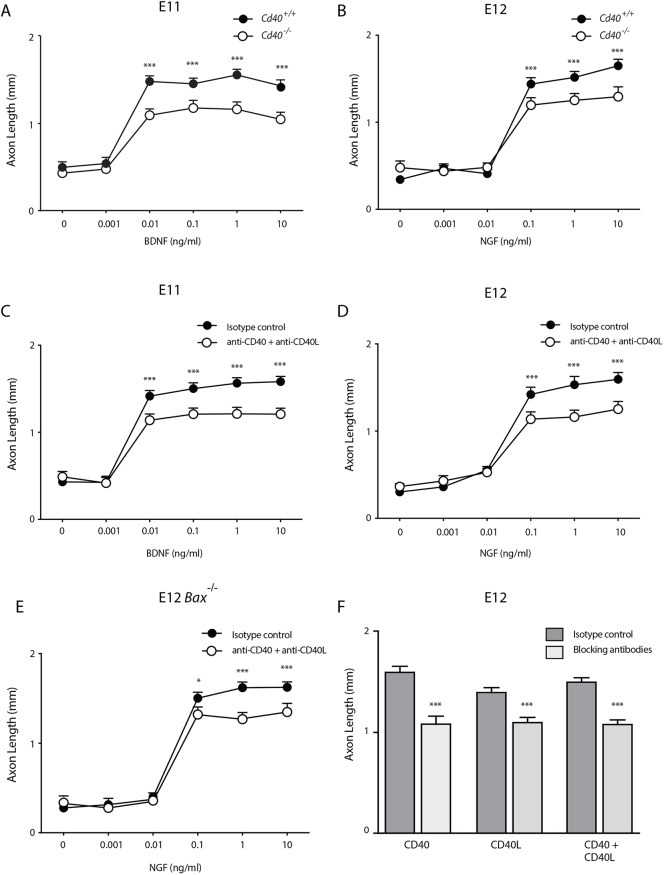
**Disruption of the putative CD40L/CD40 autocrine signalling loop decreases the axon growth response to high neurotrophin concentrations.** (A,B) Graphs of axon length of E11 (A) and E12 (B) DRG neurons of *Cd40*^−/−^ and *Cd40*^+/+^ littermates cultured for 24 h with and without BDNF (A) or NGF (B) at concentrations ranging from 0 to 10 ng/ml. (C,D) Graphs of axon length of E11 (C) and E12 (D) DRG neurons cultured for 24 h with a range of BDNF or NGF concentrations plus either function-blocking anti-CD40L and function-blocking anti-CD40 antibodies or isotype controls (each at 2 μg/ml). All cultures also received 25 µM Boc-D-FMK. (E) Axon lengths of E12 DRG BAX-deficient neurons cultured with a range of NGF concentrations together with either function-blocking anti-CD40L and function-blocking anti-CD40 antibodies or isotype controls (each at 2 μg/ml). (F) Axon lengths of E12 DRG neurons cultured with 10 ng/ml NGF and function-blocking anti-CD40L and anti-CD40 antibodies alone or in combination or with isotype controls (each at 2 μg/ml) plus 25 µM Boc-D-FMK. Data are mean±s.e.m. of >50 neurons per condition combined from three experiments of each type. **P<0.01, ***P<0.001; one-way ANOVA, statistical comparison at each BDNF or NGF concentration.

The co-expression of CD40 and CD40L by early DRG raises the possibility that axon growth may be influenced by a CD40L/CD40 autocrine signalling loop in these neurons. The similarity in axon length between *Cd40*^−/−^ and *Cd40*^+/+^ DRG neurons grown without neurotrophins (Fig. 2) suggests that CD40L/CD40 autocrine signalling does not contribute to the low level of axon growth occurring from early DRG neurons in the absence of neurotrophins. To test whether CD40L/CD40 autocrine signalling contributes to the magnitude of neurotrophin-promoted axon growth, we measured the axon lengths of DRG neurons of *Cd40*^−/−^ and *Cd40*^+/+^ embryos incubated with a range of concentrations of BDNF at E11 and NGF at E12. These dose responses showed that neurons from wild-type embryos are an order of magnitude more sensitive to BDNF at E11 than to NGF at E12 (reaching maximal responsiveness at concentrations of approximately 0.01 and 0.1 ng/ml, respectively) (Fig. 5A,B). There was no significant difference in axon length between *Cd40*^−/−^ and *Cd40*^+/+^ neurons grown with no or low concentrations of neurotrophins. However, with higher concentrations (0.01-10 ng/ml BDNF and 0.1-10 ng/ml NGF), neurons of *Cd40*^−/−^ embryos had significantly shorter axons than those of *Cd40*^+/+^ littermates (Fig. 5A,B). This suggests that disruption of a putative CD40L/CD40 autocrine loop by deleting *Cd40* impairs the growth of axons from DRG neurons stimulated with either BDNF or NGF.

An alternative way of eliminating CD40L/CD40 autocrine signalling was to prevent interaction of endogenous CD40L and CD40 in wild-type neurons by treating these neurons with function-blocking antibodies to CD40L and CD40 (McWilliams et al., 2015). In these experiments, we measured the axon lengths of E11 and E12 DRG neurons incubated with a range of concentrations of BDNF and NGF in cultures supplemented with either function-blocking antibodies or isotype control immunoglobulin. The axon growth dose responses of neurons incubated with isotype control immunoglobulin were very similar to those of *Cd40*^+/+^ neurons and the axon growth dose responses of neurons incubated with function-blocking antibodies were very similar to those of *Cd40*^−/−^ neurons. Significant differences in axon length between control-treated and function-blocking antibody-treated neurons were observed at concentrations of BDNF of 0.01 ng/ml and greater and at concentrations of NGF of 0.1 ng/ml and greater (Fig. 5C,D). The function blocking anti-CD40 and anti-CD40L antibodies were just as effective when used singularly as in combination (Fig. 5F).

To exclude the possibility that our results could have been influenced by the use of the pan-caspase inhibitor Boc-D-FMK to prevent neuronal death, we used an alternative way of preventing neuronal death in cultures that contained low levels or no neurotrophins. Neurons that lack that pro-apoptotic protein BAX fail to undergo apoptosis in the absence of NGF (Deckwerth et al., 1996). Similar results to those obtained from wild-type neurons in which death was prevented with Boc**^_^**D**^_^**FMK were obtained using neurons dissected from Bax^−/−^ embryos grown in the absence of Boc-D-FMK (Fig. 5E). Taken together, our findings support the view that a CD40L/CD40 autocrine signalling loop contributes to the magnitude of neurotrophin-promoted axon growth.

### Mechanism of action

Because early DRG neurons respond to neurotrophins, one mechanism by which CD40L might affect neurite growth is by modulating expression of NGF and BDNF receptor tyrosine kinases. However, there was no significant difference in the levels of *TrkA* (*Ntrk1*) mRNA and *TrkB* (*Ntrk2*) mRNA measured using qPCR in DRG dissected from *Cd40*^−/−^ and *Cd40*^+/+^ embryos (Fig. S3A,B) and no obvious difference in TrkA and TrkB immunocytochemistry in dissociated cultures established from *Cd40*^−/−^ and *Cd40*^+/+^ embryos (Fig. S3C,D).

**Fig. 6.**
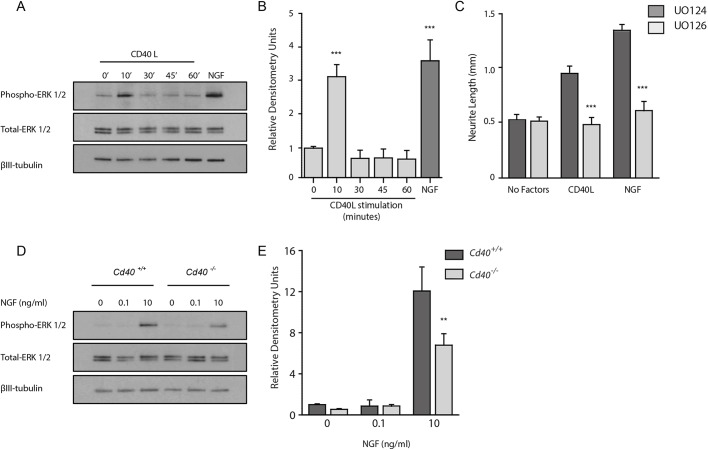
**CD40L activates ERK1/ERK2 and enhances ERK1/ERK2 activation by NGF.** (A) Representative western blots probed for phospho-ERK1/2, total ERK1/2 and βIII-tubulin of lysates of E12 DRG neurons grown for 12 h with 25 µM Boc-D-FMK and stimulated with either 100 ng/ml CD40L for the indicated times or 10 ng/ml NGF for 30 min. (B) Densitometry of phospho-ERK1/2 bands from three separate experiments. (C) Lengths of E12 DRG neurons that had been pre-incubated with 10 µM of either U0124 or U0126 for 1 h then incubated for 24 h with either no factors, 100 ng/ml CD40L or 10 ng/ml NGF. All cultures also received 25 µM Boc-D-FMK. Data are mean±s.e.m. of >50 neurons per condition combined from three experiments of each type. ***P<0.001; one-way ANOVA, statistical comparison between U0124-treated and U0126-treated cultures. (D) Representative western blots probed for phospho-ERK1/2, total ERK1/2 and βIII-tubulin of lysates of E12 DRG cultures established from *Cd40*^−/−^ and *Cd40*^+/+^ littermates grown for 12 h with 25 µM Boc-D-FMK and stimulated with either 0, 0.1 or 10 ng/ml NGF for 30 min. (E) Densitometry of phospho-ERK1/2 bands. Data are mean±s.e.m. from three experiments. **P<0.01; one-way ANOVA, statistical comparison between genotypes.

Because ERK signalling contributes to neurotrophin-promoted axon growth (Atwal et al., 2000; O'Keeffe et al., 2008) and because CD40 signalling modulates neurotrophin responsiveness (Fig. 5), we investigated whether ERK signalling contributes to CD40L-promoted axon growth. We first tested whether CD40L activates ERK1 and ERK2 in cultured DRG neurons. In these experiments, we cultured E12 DRG neurons for 12 h with the caspase inhibitor Boc-D-FMK before treating them with either CD40L or NGF as positive control. Western blotting revealed that CD40L caused rapid increases in the amounts of phospho-ERK1 and phospho-ERK2 within 10 min, which returned to basal levels within 30 min (Fig. 6A,B). Densitometry of multiple blots revealed that activation of ERK by CD40L was of similar magnitude to that NGF. These results show that CD40L causes rapid, transient activation of ERK1 and ERK2 independently of NGF.

To investigate whether ERK1 and ERK2 activation is responsible for the enhanced axon growth brought about by CD40L, we examined whether U0126, a selective MEK1 and MEK2 inhibitor that interferes with MEK1- and MEK2-dependent activation of ERK1 and ERK2 (Favata et al., 1998), could prevent CD40L-promoted axon growth. In these experiments, we plated E12 DRG neurons in NGF-free medium containing Boc-D-FMK and pre-treated these neurons for 1 h with either U0126 or the inactive analogue U0124 before adding CD40L and quantifying the axon length 24 h later. U0126, but not U0124, completely prevented CD40L-enhanced axon growth (Fig. 6C). This suggests that MEK/ERK signalling has a crucial role in mediating the effect of CD40L on axon growth.

Because the axon growth response of DRG neurons to neurotrophins is reduced in CD40-deficient neurons (Fig. 5), we investigated whether CD40 signalling affects the magnitude of ERK activation by NGF. In these experiments, DRG neurons from E12 *Cd40*^−/−^ and *Cd40*^+/+^ embryos were cultured for 12 h in the presence of Boc-D-FMK before being stimulated with 0, 0.1 or 10 ng/ml NGF for 30 min. Western blotting revealed that ERK activation occurred in response to NGF in neurons of both genotypes (Fig. 6D) but was significantly lower in neurons in CD40-deficient neurons (Fig. 6E). These results suggest that CD40 signalling contributes to NGF-promoted ERK activation.

## DISCUSSION

We have shown that CD40L is a key regulator of sensory axon growth at an early stage of development. In culture, CD40L enhances axon growth from dissociated DRG neurons independently of neurotrophins at the stage of development when DRG axons are growing to their peripheral targets. This effect is very transient, beginning when neurons start responding to BDNF and NGF. It is virtually over by E13, when many axons have reached their targets and have become NGF-dependent. In common with other members of the TNFSF that regulate axon growth, either by forward or reverse signalling mechanisms (Desbarats et al., 2003; Gavalda et al., 2009; Gutierrez et al., 2008, 2013; Kisiswa et al., 2013, 2017; McWilliams et al., 2015; O'Keeffe et al., 2008; Wheeler et al., 2014), and in marked contrast to neurotrophins (Davies, 2003), CD40L has no effect on neuronal survival.

Importantly, we observed a phenotype that matched these *in vitro* findings in mice lacking CD40, the receptor for CD40L. During the period of development when DRG axons are growing toward and start to reach their peripheral targets, spinal nerves were significantly shorter in *Cd40*^−/−^ embryos compared with *Cd40*^+/+^ littermates. Highly significantly reductions in thoracic nerve length in *Cd40*^−/−^ embryos compared with *Cd40^+/+^* embryos were observed at each age studied (E11, E12 and E13) and there were also significant reductions in hind limb plexus complexity in *Cd40*^−/−^ embryos at E12 and E13. These *in vivo* observations confirm the physiological relevance of our *in vitro* findings. However, although the relative reduction in thoracic nerve length in *Cd40*^−/−^ embryos was proportionately greater at E12 than at E13, the relative reduction in hind limb plexus complexity was proportionately greater at E13 than at E12. Whether these age-relative regional differences in relative magnitude of the significant effects of the *Cd40* null mutation reflect statistical variation or possibly some degree of compensation in the thoracic nerves of *Cd40*^−/−^ embryos is unclear.

The early developmental effect of CD40L on sensory axon growth is in marked contrast to the actions of other members of the TNFSF that regulate sensory and sympathetic axon growth in the developing PNS. In all studies to date, TNFSF members acting by either forward or reverse signalling influence axon growth much later in development, well after neurons have become dependent on neurotrophins for survival when their axons are ramifying in and refining terminations in their targets (Desbarats et al., 2003; Gavalda et al., 2009; Gutierrez et al., 2008, 2013; Howard et al., 2018; Kisiswa et al., 2013, 2017; McWilliams et al., 2015; Nolan et al., 2014; O'Keeffe et al., 2008; Wheeler et al., 2014). In addition to regulating axon growth by either forward or reverse signalling mechanisms, TNFSF members also regulate the extent of axon branching *in vitro* (Gavalda et al., 2009; Gutierrez et al., 2008, 2013; Howard et al., 2018; Kisiswa et al., 2013; McWilliams et al., 2015; O'Keeffe et al., 2008). This in turn influences target field innervation density *in vivo* (Howard et al., 2018; Kisiswa et al., 2013; McWilliams et al., 2015; O'Keeffe et al., 2008; Wheeler et al., 2014). In contrast, we have shown that CD40L has no effect on DRG axon branching. This may reflect the early stage of development during which CD40L acts, a stage in which DRG neurons are normally bipolar and unbranched.

The very early effect of CD40L on sensory axon growth raises the possibility that CD40L plays a role in guiding sensory axons to their targets, as has been demonstrated for specific target-derived sensory axon attractants (Lumsden and Davies, 1983, 1986). However, this seems to be unlikely because the gross anatomy of sensory fibres in early *Cd40*^−/−^ embryos appears to be normal compared with *Cd40*^+/+^ littermates. It therefore appears that the principal role of CD40L in the developing sensory nervous system is to accelerate the growth of sensory axons to their targets without influencing the direction in which they grow.

In addition to the uniquely early stage of development at which CD40L acts on sensory neurons, CD40L differs from other TNFSF members in the way it regulates axon growth in the developing PNS. Other TNFSF members do not affect axon growth in the absence of neurotrophin signalling, but selectively modulate the axon growth response of neurons to neurotrophins. Either they enhance the neurotrophin axon growth response (Kisiswa et al., 2013; McWilliams et al., 2015; O'Keeffe et al., 2008) or impair this response (Gavalda et al., 2009; Gutierrez et al., 2008, 2013; Howard et al., 2018). In contrast, CD40L is able to promote the growth of early sensory axons in the absence of neurotrophins.

Although CD40L is able to promote axon growth independently of neurotrophins in early sensory neurons, CD40L/CD40 autocrine signalling is also able to modulate the axon growth response of these neurons to neurotrophins. Many neurons co-express a TNFSF member and the TNFRSF(s) member to which it binds, and influence the neurotrophin axon growth response by engaging an autocrine signalling loop (Gavalda et al., 2009; Gutierrez et al., 2008; McWilliams et al., 2015; O'Keeffe et al., 2008). Early sensory neurons co-express CD40 and CD40L, and disrupting a putative CD40L/CD40 autocrine loop in very low-density cultures significantly reduces the axon growth response to BDNF and NGF. This suggests that CD40L/CD40 autocrine signalling makes the neurons significantly more responsive to the axon growth-promoting ability of these neurotrophins. This enhanced neurotrophin responsiveness may be part of the reason why early DRG nerve fibres are shorter in *Cd40*^−/−^ embryos compared with *Cd40*^+/+^ littermates. In addition, the synthesis of CD40L in peripheral target tissues may also enhance axon growth *in vivo* by acting directly on the growing axons. The expression of CD40 along the axons of early sensory neurons suggests that these neurons are potentially capable of responding to target-derived CD40L. We are unable to comment on the relative contributions of CD40L/CD40 autocrine signalling and target-derived CD40L to early sensory axon growth *in vivo*. However, the shift in the expression of *Cd40l* mRNA from neurons to targets in early development suggests that autocrine signalling predominates earlier than target-derived CD40L.

A clear difference between our current results and published studies on CD40 is the direction of signalling. A variety of experimental approaches have demonstrated that CD40-activated CD40L-mediated reverse signalling regulates the growth of sympathetic axons, hippocampal pyramidal neuron axons, medium spiny neuron dendrites and hippocampal pyramidal neuron dendrites (Carriba and Davies, 2017; McWilliams et al., 2015). In contrast, our finding that soluble CD40L enhances sensory axon growth in a CD40-dependent manner and our demonstration that soluble CD40-Fc, which activates reverse signalling, has no effect on sensory axon growth suggests the operation of CD40L-activated CD40-mediated forward signalling in early sensory neurons. Although CD40 is the principal receptor for CD40L, it is recognized that CD40L can bind to other receptors, such as members of the integrin family (Michel et al., 2017). However, the complete elimination of the CD40L-promoted axon growth response in CD40-deficient early sensory neurons suggests that CD40 is the relevant receptor.

The direct neurotrophin-independent effects of CD40L on early sensory axon growth and its indirect effect of modulating the sensitivity of the neurons to the axon growth-promoting effects of neurotrophins appears to be mediated largely by the well-recognised axon growth-promoting pathway that includes ERK activation (Atwal et al., 2000; O'Keeffe et al., 2008). CD40L causes a marked activation of ERK1/ERK2 that is of similar magnitude to that promoted by neurotrophins, and pharmacological blockade of ERK1/ERK2 activation eliminates the axon growth-promoting effect of CD40L. The efficiency with which neurotrophins promote ERK1/ERK2 activation is markedly and significantly reduced in neurons cultured from *Cd40*^−/−^ embryos. How CD40 signalling affects ERK activation is an intriguing question for future research.

In summary, we have shown that CD40L-activated CD40-mediated forward signalling is a novel physiological regulator of axon growth in the developing nervous system. In striking contrast to late developmental action of other TNFSF members that regulate axon growth, CD40L acts very early in neuronal development. The transient effect of CD40L on DRG neurons declines markedly as their axons reach their targets. In future work it will be informative to see whether CD40-mediated forward signalling operates elsewhere in the developing nervous system. It will also be interesting to ascertain whether the early effects of CD40L on sensory neurons influence future somatosensory function, as this may provide insights for the selection-advantage of this particular developmental role of CD40L. It will also be interesting to elucidate and compare the downstream events from CD40 and CD40L that mediate the effects of forward and reverse signalling on the growth of neural processes.

## MATERIALS AND METHODS

### Animals

This study was conducted on tissues obtained from CD1 wild-type mice (*Mus musculus*), *Cd40* null mutant mice in a C57BL6/J background that were purchased from The Jackson Laboratory and *Bax* null mutant mice (a gift from Stanley Korsmeyer, Harvard Medical School, Boston, MA, USA). The mutant mice were back-crossed for at least 10 generations into a CD1 background before undertaking any of the current experimental work. *Cd40*^+/−^ mice were crossed to generate *Cd40*^+/+^ and *Cd40*^−/−^ littermates. Breeding, housing and genotyping was approved by the Cardiff University Ethical Review Board and was performed within the guidelines of the Home Office Animals (Scientific Procedures) Act, 1986.

### Neuron culture

DRG were dissected from embryonic CD1 mice. The ganglia were trypsinized and plated at very low density (∼500 neurons per dish) in poly-ornithine/laminin-coated 35 mm or 4-well tissue culture dishes (Greiner Bio-One) in serum-free Hams F14 medium (Davies et al., 1993) supplemented with 0.25% Albumax I (Life Technologies).

Neuronal survival was estimated by counting the number of attached neurons within a 12×12 mm grid in the centre of each dish 2 h after plating and again after 24 h, and expressing the 24 h count as a percentage of the 2 h count. Analysis of neurite length was carried out by labelling the neurons at the end of the experiment with the fluorescent vital dye Calcein-AM (Life Technologies). For every condition in each experiment, images of at least 50 neurons were digitally acquired by fluorescence microscopy and analyzed to obtain total neurite length (Gutierrez and Davies, 2007). Statistical analyses were performed using a one-way ANOVA with Bonferroni-Holm post hoc test. Pair-wise comparisons were made using Student's *t*-test. NGF, CD40L, CD40-Fc and Fc control protein were obtained from R&D Systems. The MEK1 and MEK2 inhibitor U0126 and inactive analogue U0124 were purchased from Sigma-Aldrich. Where indicated, the culture medium was supplemented with the caspase inhibitor Boc-D-FMK (Calbiochem) to prevent neuronal apoptosis in the absence of neurotrophins.

### Immunocytochemistry

The culture medium was gently aspirated and the cultures were washed with PBS at 37°C. Cells were fixed with either ice-cold methanol (MeOH) for 5 min or freshly made 4% paraformaldehyde (Sigma-Aldrich) in 0.12 M phosphate buffer (pH 7.2) for 12 min. The fixative was removed and, after washing with PBS, the cultures were blocked for ∼1 h at room temperature in 5% bovine serum albumin (BSA) containing 0.2% Triton X-100 (Sigma-Aldrich). The cultures were then incubated overnight at 4°C with primary antibody in PBS containing 1% BSA (Sigma-Aldrich) and were gently agitated on an orbital shaker. After extensive washing in PBS, the cultures were incubated with fluorophore-conjugated secondary antibody (Alexa Fluor 488/546, Thermo Fisher Scientific) in 1% BSA for 1 h in the dark at room temperature. Following serial washes in PBS, the nuclei were counterstained with DAPI (1:10,000, Life Technologies) for 5 min.

The cultures were imaged using either a Zeiss Axiovert 200 Inverted Fluorescence microscope or a Zeiss LSM 710 Confocal Laser Scanning Microscope using Zen software (Zeiss). The following primary antibodies were used: rabbit polyclonal anti-CD40L (1:300, ab2391, Abcam), rabbit polyclonal anti-CD40 (1:300, ab13545, Abcam), mouse monoclonal anti-CD40 (1:300, ab91075, Abcam) and mouse monoclonal anti-βIII-tubulin (1:500, MAB1195, R&D Systems).

### Immunohistochemistry

E12 embryos were fixed for 1 h in freshly made 4% PFA, washed in PBS, and cryoprotected in 30% sucrose in PBS. Embryos were embedded in Tissue-Tek OCT Compound (Sakura Finetek Europe), in Peel-A-Way moulds (Ted Pella). Embryos were oriented for sectioning and frozen by slow immersion in isopentane cooled with dry ice and sectioned using a Leica CM 1850UV cryostat. Sections were mounted onto Xtra-Adhesive slides (Surgipath). For immunohistochemical localization of proteins, slides were washed with PBS, blocked with 5% BSA containing 0.1% Triton X-100, and incubated overnight with rabbit polyclonal anti-CD40 (1:300, ab13545, Abcam) in PBS containing 1% BSA. After extensive washing with PBS, the slides were incubated with fluorophore-conjugated secondary antibody (1:500, Alexa Fluor 546, Thermo Fisher Scientific) in 1% BSA for 1 h in the dark and imaged using a Zeiss LSM 710 Confocal Laser Scanning Microscope using Zen software.

### Analysis of DRG peripheral fibres *in vivo*

Analysis of sensory fibre growth *in vivo* was carried out using iDISCO preparations (Renier et al., 2014) of E11, E12 and E13 *Cd40*^+/+^ and *Cd40*^−/−^ embryos. Briefly, embryos were fixed in 4% paraformaldehyde for 24 h and serially dehydrated in methanol/phosphate buffered saline (PBS). The samples were then bleached overnight in chilled 5% H_2_O_2_ to reduce tissue auto-fluorescence before being serially rehydrated in methanol/PBS containing 0.2% Triton X-100. The embryos were incubated in blocking solution (6% donkey serum, 20% DMSO, 0.2% Triton X-100, 0.3 M Glycine in PBS) for 72 h at 37°C. After washing with PBS containing 0.2% Tween-20 and 10 mg/ml heparin (PTwH), the embryos were incubated with βIII-tubulin antibody (1:300, MAB1195, R&D Systems) in PTwH containing 5% DMSO and 3% donkey serum for 72 h at 37°C. Following extensive washing in PTwH, the samples were incubated with donkey anti-mouse 546 Alexa Fluor secondary antibody (1:300, A10036, Life Technologies) in PTwH plus 3% donkey serum for 72 h at 37°C. After further washing in PTwH, samples were cleared by overnight incubation in tetrahydrofuran, followed by dichloromethane treatment for 15 min. The samples were placed in dibenzyl ether (DBE) until clear and imaged while submerged in DBE in 3D-printed slide chambers using a Zeiss LSM710 confocal microscope. The *Cd40*^−/−^ data are expressed as a percentage of the mean of *Cd40*^+/+^ data. All imaging and quantification was performed blind with genotypes determined after quantification.

### RT-qPCR

The levels of *Cd40l*, *Cd40*, *TrkA* and *TrkB* mRNAs were quantified by qPCR relative to a geometric mean of mRNAs for the housekeeping enzymes glyceraldehyde phosphate dehydrogenase (*Gapdh*), succinate dehydrogenase (*Sdha*) and hypoxanthine phosphoribosyltransferase 1 (*Hprt1*). Total RNA was extracted from dissected DRG or hind limb buds using the RNeasy Micro extraction kit (Qiagen) and 5 μl was reverse transcribed for 1 h at 45°C using the AffinityScript kit (Agilent) in a 25 µl reaction according to the manufacturer's instructions. Then 2 µl of cDNA was amplified in a 20 µl reaction volume using Brilliant III ultrafast qPCR master mix reagents (Agilent). PCR products were detected using dual-labelled (FAM/BHQ1) hybridization probes specific to each of the cDNAs (Eurofins). The PCR primers were: *Cd40l* forward, 5′-TGGATCTGAGAGAATCTTACT-3′ and reverse, 5′-AGTCACGTTGACAAACAC-3′; *Cd40* forward, 5′-CTTTGGAGTTATGGAGATG-3′ and reverse, 5′-ATGACTGATTGGAGAAGA-3′; *TrkA* forward, 5′-CTGTGTCCATCACATCAA-3′ and reverse, 5′-GAAGGTTGTAGCACTCAG-3′; *TrkB* forward, 5′-AAGTTTGGCATGAAAGGC-3′ and reverse, 3′-ATTGGAGATGTGGTGGAG-3′; *Gapdh* forward, 5′-GAGAAACCTGCCAAGTATG-3′ and reverse, 5′-GGAGTTGCTGTTGAAGTC-3′; *Sdha* forward, 5′-GGAACACTCCAAAAACAG-3′ and reverse, 5′-CCACAGCATCAAATTCAT-3′; *Hprt1* forward, 5′-TTAAGCAGTACAGCCCCAAAATG-3′ and reverse, 5′-AAGTCTGGCCTGTATCCAACAC-3′. Dual-labelled probes were: *Cd40l*, 5′-FAM-CGGCAAATACCCACAGTTCCT-BHQ1-3′; *Cd40*, 5'-FAM-CCACTGAGACCACTGATACCG-BHQ1-3′; *TrkA*, 5′-FAM-CGCCAGGACATCATTCTCAAGT-BHQ1-3′; *TrkB*, 5′-FAM-CGGTCATCAGCAACGACGAT-BHQ1-3′; *Gapdh*, 5′-FAM-AGACAACCTGGTCCTCAGTGT-BHQ1-3; *Sdha*, 5′-FAM-CCTGCGGCTTTCACTTCTCT-BHQ1-3, *Hrpt1*, FAM-TCGAGAGGTCCTTTTCACCAGCAAG-BHQ1-3′. Owing to extremely low level expression of *Cd40l* mRNA, it was necessary to use a nested amplification protocol, by pre-amplifying cDNA for eight cycles with the forward primer, 5′-AAGGACTCTATTATGTCTACAC-3′ and reverse primer, 5′-GATGAGAAGCCAACTCTG-3′ before amplification with the *Cd40l* primer and probe set as described above.

Forward and reverse primers were used at a concentration of 150 nM and dual-labelled probes were used at a concentration of 300 nM. PCR was performed using the Mx3000P platform (Agilent) using the following conditions: 45 cycles of 95°C for 12 s and 60°C for 35 s. Standard curves were generated for each cDNA for every RT-PCR run, by using serial fivefold dilutions of reverse transcribed mouse adult spleen total RNA (Zyagen). Relative mRNA levels were quantified in four separate sets of dissected DRG and hind limb buds for each embryonic age. Primer and probe sequences were designed using Beacon Designer software (Premier Biosoft).

### Quantification of DRG neuron numbers *in vivo*

The number of neurons in level-matched L4 DRG of E12 *Cd40*^−/−^ and *Cd40*^+/+^ embryos was quantified using a combined stereological and confocal approach (Howell et al., 2002; Yoshikawa et al., 2007). The DRG were fixed in 4% PFA and cryoprotected in 30% sucrose before being serially sectioned at 10 μm. Sections were blocked in 5% BSA (Sigma-Aldrich) for 1 h and were then incubated overnight at 4°C with rabbit monoclonal anti-NeuN (1:500, ab177487, Abcam) and mouse monoclonal anti-βIII-tubulin (1:500, MAB1195, R&D Systems) in PBS containing 1% BSA. After gentle washing with PBS, the sections were incubated with fluorophore-conjugated secondary antibody (1:500, Alexa Fluor 488/546, Thermo Fisher Scientific) in 1% BSA for 1 h in the dark at room temperature. Imaging was performed using a Zeiss LSM710 confocal microscope and the number of immunoreactive neurons were counted from the level-matched lumbar DRGs of each genotype. Using the 10 μm serial sections containing whole L4 DRGs at E12, the areas of DRG in each section were measured and the DRG volume was calculated using Zen Black imaging software (Zeiss).

### Immunoblotting

DRG neurons cultured at high density in 96-well plates (>5000 cells/well) were lysed in ice-cold RIPA lysis buffer supplemented with protease and phosphatase inhibitor cocktail mix (Sigma-Aldrich) and insoluble debris was removed by centrifugation at 200 ***g***. Equal quantities of protein were run on pre-cast 4-20% SDS-PAGE gels (Bio-Rad) and were transferred to PVDF membranes using a TransBlot Turbo Apparatus (Bio-Rad), which were then incubated with blocking solution for 1 h at room temperature (5% non-fat dry milk in PBS with 0.1% Tween-20; PBS-T). After washing with PBS-T the blots were probed overnight at 4°C with rabbit anti-phospho-ERK1/ERK2 (1:1000, ab9101, Cell Signaling Technologies), rabbit anti-total ERK1/ERK2 (1:1000, ab9102, Cell Signaling Technologies) or mouse anti-β-III-tubulin (1:5000, MAB1195, R&D Systems). Bound primary antibodies were visualized with HRP-conjugated anti-mouse or anti-rabbit secondary antibodies (1:2000, W4021 or W4011, respectively, Promega) and Immunocruz Luminol reagent (Santa Cruz Biotechnology) and Amersham Hyperfilm ECL (GE Healthcare Life Sciences). Densitometry was carried out using Image Studio Lite (Li-cor Biosciences). The levels of phospho-ERK1 and phospho-ERK2 were normalized to the levels of total ERK1 and ERK2.

## Supplementary Material

Supplementary information
