# Supplementary material for: CD40 forward signalling is a physiological regulator of early sensory axon growth
Source: Development. 2019 Sep 16;146(18):dev176495. doi: 10.1242/dev.176495 (PMC6765180; doi:10.1242/dev.176495)
Supplement: Supplementary information [file develop-146-176495-s1.pdf]

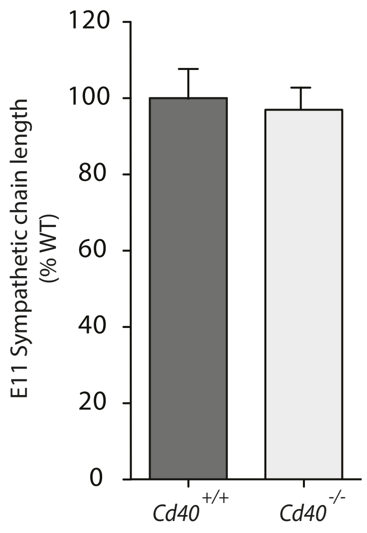

Figure S1 No significant difference in sympathetic chain length between *Cd40*$^{-/-}$ and *Cd40*$^{+/+}$ embryos. Bar chart of the length of the part of the paravertebral sympathetic chain between the rostral tip of the SCG and the 6th thoracic DRG in the same E11 *Cd40*$^{-/-}$ and *Cd40*$^{+/+}$ embryos that were used to measure sensory nerve length. The data are normalised to 100% for *Cd40*$^{+/+}$ embryos.

Development • Supplementary information

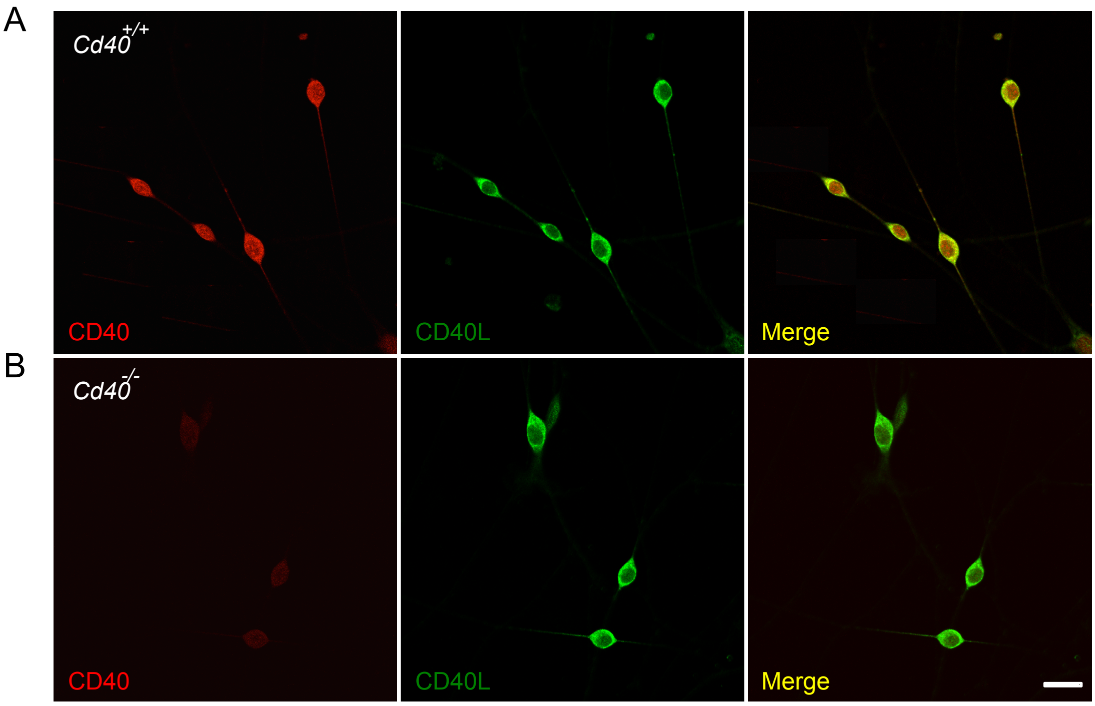

Figure S2 CD40 immunofluorescence is eliminated in neurons cultured from *Cd40*$^{-/-}$ embryos. Representative photomicrographs of E12 DRG neuron cultures established from *Cd40*$^{+/+}$ (A) and *Cd40*$^{-/-}$ (B) embryos double labeled with anti-CD40 and anti-CD40L. Scale bar, 20 µm.

Development • Supplementary information

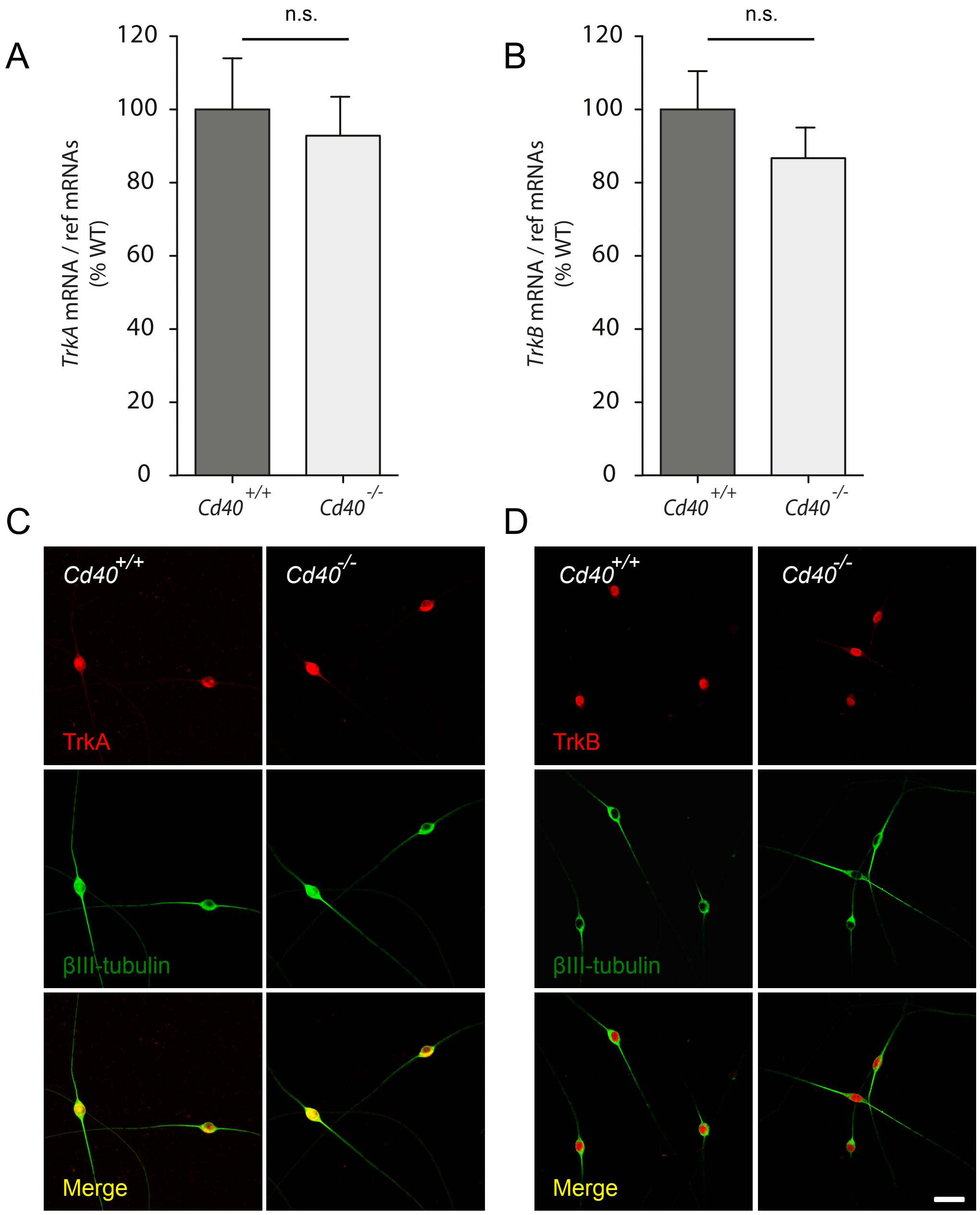

Figure S3. No significant differences in DRG Trk expression between *Cd40*$^{-/-}$ and *Cd40*$^{+/+}$ mice. (A, B) Bar charts of the levels of *TrkA* mRNA (A) and *TrkB* mRNA (B) in E12 DRG of *Cd40*$^{-/-}$ and *Cd40*$^{+/+}$ embryos relative to the geometric mean of reference mRNAs for glyceraldehyde phosphate dehydrogenase, succinate dehydrogenase and hypoxanthine phosphoribosyltransferase-1. The data are normalized to 100% in DRG of *Cd40*$^{+/+}$ embryos. Mean ± s.e.m. of 3 independent measurements. (C, D) Representative photomicrographs of E12 DRG neuron cultures established from *Cd40*$^{-/-}$ and *Cd40*$^{+/+}$ embryos double labeled with anti-βIII tubulin and either anti-TrkA (C) or anti-TrkB (D). Scale bar, 20 μm.

Development • Supplementary information
